# A new cellular type in invertebrates: first evidence of telocytes in leech *Hirudo medicinalis*

**DOI:** 10.1038/s41598-017-13202-9

**Published:** 2017-10-19

**Authors:** Laura Pulze, Nicolò Baranzini, Rossana Girardello, Annalisa Grimaldi, Lidia Ibba-Manneschi, Enzo Ottaviani, Marcella Reguzzoni, Gianluca Tettamanti, Magda de Eguileor

**Affiliations:** 1 University of Insubria, Department of Biotechnology and Life Sciences, 21100 Varese, Italy; 2 University of Florence, Dipartimento di Medicina Sperimentale e Clinica, 50134 Firenze, Italy; 3 University of Modena & Reggio Emilia; Department of Life Sciences, 41125 Modena, Italy; 4 University of Insubria, Department of Surgical and Morphological Sciences, 21100 Varese, Italy

## Abstract

Telocytes, a peculiar cell type, were recently found in vertebrates. Hence this cell system has been reported as ubiquitous in the bodies of mammals and interpreted as an important player in innate immunity and tissue regeneration, it is reasonable to look for it also in invertebrates, that rely their integrity solely by innate immunity. Here we describe, at morphological and functional level, invertebrate telocytes from the body of leech *Hirudo medicinalis* (Annelida), suggesting how these cells, forming a resident stromal 3D network, can influence or participate in different events. These findings support the concepts that leech telocytes: i) are organized in a cellular dynamic and versatile 3D network likewise the vertebrate counterpart; ii) are an evolutionarily conserved immune-neuroendocrine system; iii) form an immuno-surveillance system of resident cells responding faster than migrating immunocytes recruited in stimulated area; iv) communicate with neighbouring cells directly and indirectly, via cell-cell contacts and soluble molecules secreted by multivesicular bodies; v) present within neo-vessels, share with immunocytes the mesodermal lineage; vi) are involved in regenerative processes. In conclusion, we propose that HmTCs, integrating so different functions, might explain the innate immune memory and can be associated with several aged related diseases.

## Introduction

Telocytes (TCs) are interstitial (stromal) cells recently described in humans and rodents and located in cavitary and non cavitary organs such as heart, gut, gallbladder, uterus, lung, pancreas, mammary gland and skeletal muscles^1–9^. The TCs present in various types of tissues are strategically distributed among resident cells, close to the capillaries and the nerve endings. They are always characterized by a small cell body, containing few organules and the nucleus, and very thin, long processes named telopods (TPs), parameters that are considered the ultrastructural peculiar traits of these cells. Each TP, long and convoluted, is moniliform showing thin tubes (podomers) alternated with dilations (podoms). TCs may contact with each other and with virtually all type of cells forming a close network^10,11^. The cross-talk among TCs and the other cells, is thought to influence various processes directly via gap junctions, and/or indirectly via the release of extracellular vesicles (multivesicular bodies, exosomes)^12–17^. TCs are hypothesized as an extensive intercellular information transmission system able to modulate homeostasis, stem cell activity, and other functions operating as a primitive nervous system and/or as a immunomodulator at the cellular level^8,13,17^. These cells are described as completely different from fibroblasts, fibroblast-like cells, myofibroblasts and/or mesenchymal cells, not only morphologically, but also in terms of immunocytochemical responses. Moreover, while there is a morphological ultrastructural hallmark for identifying TCs, their immunophenotype is stated as variable within different organs/tissues, according to oxidative status of cell environment and in relation to aging/pathological conditions^18–22^.

Several authors considered that double-positive immunostaining with CD34/PDGFRα and CD34/vimentin might be useful in identifying these cells^23–27^. Sometimes and temporarily TCs may also express c-kit, Sca-1 and Oct-4 which are stem cell markers^2,8,21^.

TCs have been proposed to be actors in the transduction of inputs from neurons; as cells joining the stem cells in injured tissues in humans^22,28^; as connectors of immune cells during infectious and inflammatory processes^18,29–31^; as intercellular mediators during the morphogenesis of heart^32^ and as important players in immune responses^8^. Owing to the multifaceted biological function of TCs many questions remain opened about their relationship with other cells and concerning their critical role in normal and in pathological conditions.

Hence this cell system has been reported as ubiquitous in vertebrate bodies and has been interpreted as an important player in innate immunity and tissue regeneration, it is reasonable to look for it in invertebrates too, bearing in mind that they rely their integrity solely by innate immunity. Aside from numerous vertebrate morpho-functional studies on TCs, there are no data about the presence of these cells in invertebrates.

Here we describe, at morphological and functional level, TCs from invertebrates, hypothesizing the function of this 3D network in the medicinal leech *Hirudo medicinalis* (Annelida, Hirudinea) body. It should be simpler to understand how TCs influence or participate in different events and how they are involved in intercellular cross-talk, in an invertebrate animal model than in vertebrates characterized by a complex body plan. Leeches have a reduced dimension and a relative anatomical simplicity but share with vertebrates the complexity of immunological mechanisms and wound-healing processes^33–45^. Thus we take advantage of the fact that biological responses, cell types, cellular mechanisms, and molecules are surprisingly similar to those occurring in vertebrates, suggesting a remarkable evolutionary conservation in animal kingdom.

## Results

### Leech body organization

**Figure 1:**
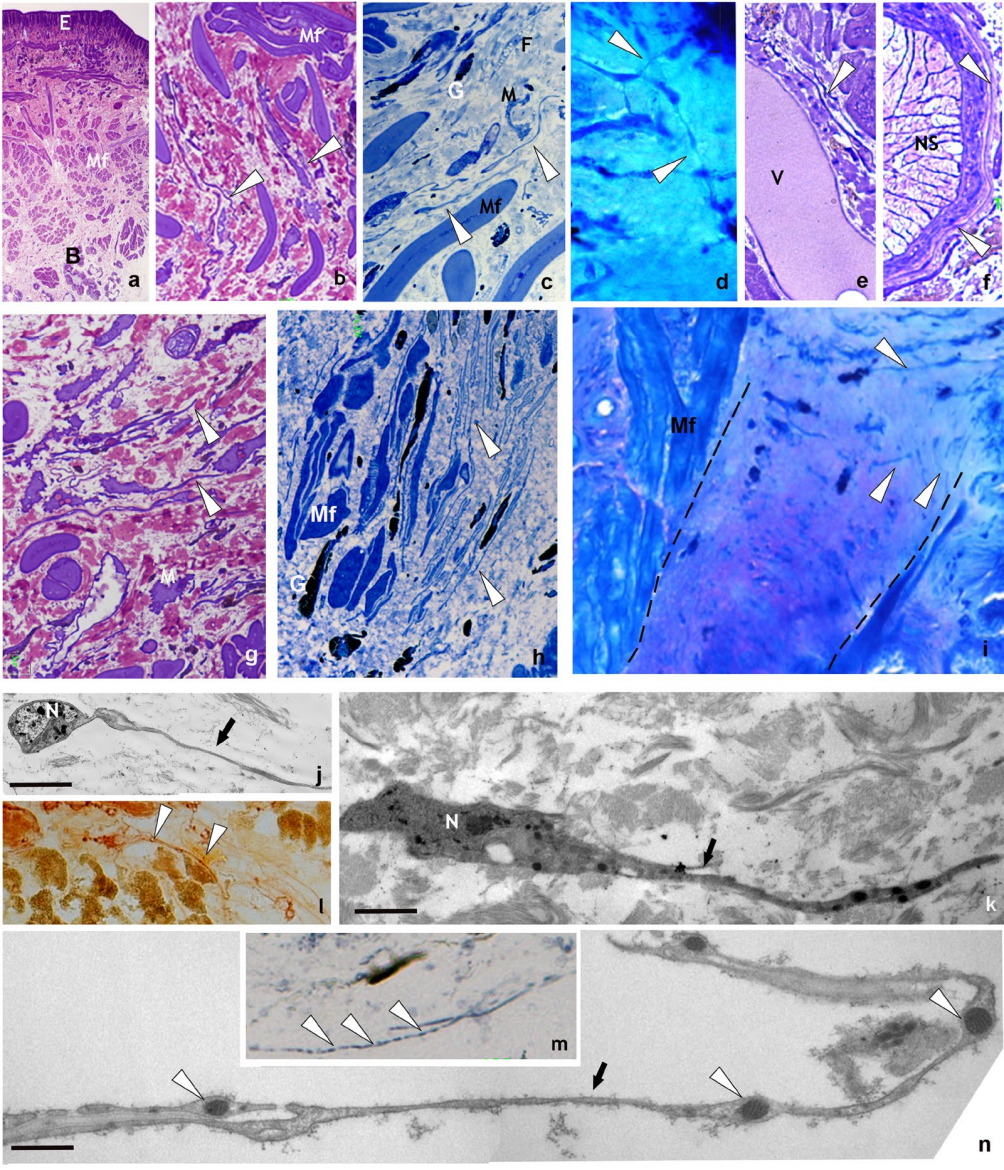
Unstimulated and stimulated *H. medicinalis*: optical and TEM microscopy. (**a**–**f**) Light microscopy, semi-thin sections of healthy (unstimulated) leech. (**a**) General view of cross sectioned body of leech (Crystal violet staining). Under the epithelium (E), the body wall is mainly composed of tightly packed helical muscle fibers (Mf) embedded in a scarce and loose connective tissue. Under the longitudinal layer of muscle fibers the botryoidal tissue is visible (B). (**b**) Detail of muscular body wall. Among muscle fibers (Mf), embedded in the loose connective tissue, interstitial cells showing telocyte phenotype are visible (white arrowheads). (**c**) Specimen stained with methylene blue, which is considered the best dye in identifying telocyte feature. In the connective tissue HmTCs, showing small cell bodies and thin long cellular processes (white arrowheads) are easily recognizable in respect to granulocytes (G), fibroblasts (F) and macrophages (M). (**d**) Specimen May-Grunwald Giemsa. HmTCs (arrowheads) in close contact by their tubular long cytoplasmic processes are embedded in the loose connective tissue. (**e**,**f**) Specimens Toluidine blue stained. HmTCs’ telopodes (arrowhead) surround a small vessel (V) and ganglia (nervous system-NS). (**g**–**i**) Semi-thin sections of leeches stimulated by LPS injection (**g**,**h**) or subjected to surgical lesion (**i**). In the specimens stained with crystal violet (**g**) and with methylene blue (**h**) telocytes (white arrowheads), wide distributed in the connective tissue, among muscle fibers (Mf) and granulocytes (G), are more numerous than controls. In leech surgically lesioned (specimen stained with May-Grunwald Giemsa) (**i**), the outlined wedge-shaped regenerating tissue is characterized by a large amount of newly synthesized connective tissue infiltrated with proliferating (expressing stemness factors) telocytes (arrowheads). (Mf: muscle fibers). (**l**,**m**) Cryosections of leech body wall. HmTCs (arrowheads) are Nonspecific esterase+ (NSE+) (**l**) and Succinic dehydrogenase+ (SDH+) (**m**). Nonspecific esterase reaction is linked to microsomal and lysosomal activity while SDH reaction is attributable to mitochondrial activity. Intense spotted SDH staining evidences mitochondria localized in podoms (arrowheads). (**j**,**k**,**n**) Ultrastructural analysis (TEM) confirm the telocyte phenotype characterized by spindle-shape cell body and long and thin telopods (arrows). Note the localization of mitochondria within podoms (**n**) (arrowheads) that validates the SDH moniliform positivity (**m**). Scale bars. j: 2 μm; k: 0.9 μm; n: 2.2 μm.

The body wall of *H. medicinalis* consists of a monolayered epithelium enveloping a thick muscular wall made of grouped helical muscle fibers circularly, obliquely and longitudinally organized. The muscular cutaneous sac is separated from the gut by loose connective tissue, in which multifunctional botryoidal tissue (formed by cluster of large granular cells intercalated with small and flattened endothelial cells) and vasofibrous tissue cells are embedded (Fig. 1 panel a)^34,37,41,46–48^. The connective tissue, showing small and few collagen fibers, is occupied by a reduced number of resident cells such as fibroblasts, macrophages, vasofibrous granular cells associated with translucent vasocentral cells. All these cell types can be detected and identified due to their peculiar phenotypes that distinguishes one from the other.

### Identifying and characterizing *H. medicinalis* Telocyte Cells (HmTCs)

HmTCs were present among the different types of cells mentioned above but, up to now, overlooked because of their scale down dimensions and because they did not lie entirely in the same plane of investigated section (Fig. 1 panels b–i). HmTCs are interstitial cells recognized for their small irregular or spindle-shaped cell body (from 3.5 μm up to 6 μm) from which extremely long, thin and convoluted telopods (HmTPs) (up to 200 nanometers wide and up to 100 micrometers long) originated. These long thin cytoplasmic processes are made of tubes (podomers) and enlarged zones (podoms) (Fig. 1 panels j,k,n). These cells showed, in addition to the peculiar shape, an affinity for those vital dyes commonly used to characterize vertebrate TCs [crystal violet (Fig. 1 panel b), methylene blue (Fig. 1 panel c), Giemsa (Fig. 1 panel d), Toluidine blue (Fig. 1 panels e,f) staining]. After staining HmTCs were readily recognizable near botryoidal tissue, in the vicinity of capillaries (Fig. 1 panel e), among helical muscle fibers (Fig. 1 panel b,c,g,h), close to the gut and ganglia (Fig. 1 panel f).

When leeches are surgically injured or LPS injected, they respond to stimuli showing the same sequence of events described for vertebrates. Wound healing in leech is defined by inflammation step that is followed by granulation tissue stage (including wound closure and restoration, angiogenesis and fibroplasia) and by the remodelling of newly-formed tissues^36–45,47–49^. The initial inflammatory response is characterized by a massive increase in the number of immune cells such as macrophage-like, NK-like, granulocytes^33,35,36,39,43,48^ and HmTCs (Fig. 1 panels g–i).

#### Electron microscopy

**Figure 2:**
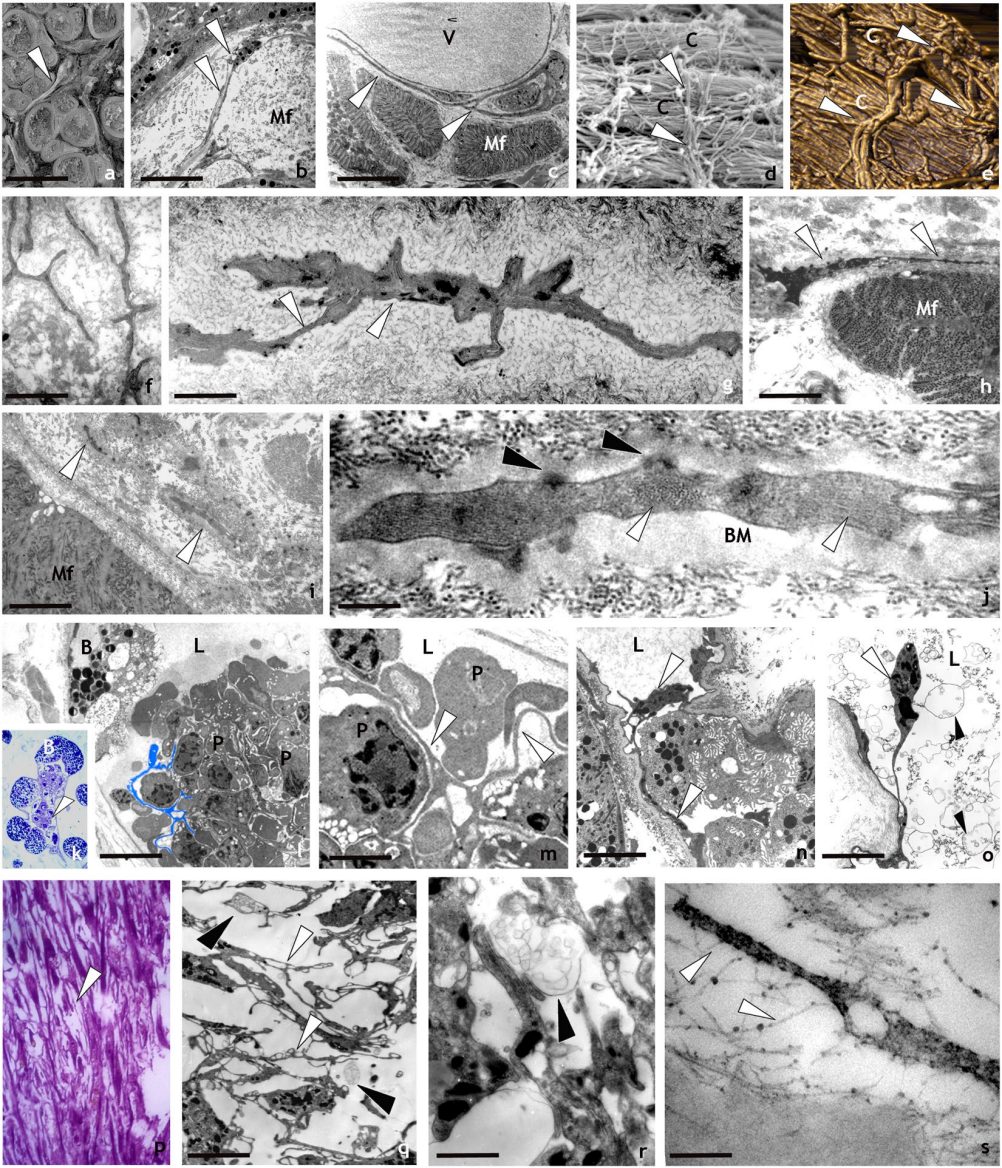
Spatial organization and behaviour of HmTCs after activation by LPS injection or surgical lesion revealed by optical, SEM, AFM and TEM analysis. (**a**–**j**) Ultrastructure: -SEM (**a**,**d**), TEM (**b**,**c**) and AFM (**e**) images of leech body wall: HmTCs (arrowheads) can be in close contact with muscle fibres (Mf), embrace vessels (V) with their telopodes or are orthogonally placed in respect to collagen bundles (**c**) as visible both in SEM and AFM photographs (**d**,**e**). Scale bars. b: 4 μm; c: 3 μm; f: 3 μm; g: 1.4 μm; h: 2 μm; i: 1.7 μm; j: 0.2 μm; l: 4 μm; m: 1 μm; n: 3 μm; o- 3 μm; q: 3 μm;r: 1.4 μm; s: 0.25 μm. (**f**–**j**) Thin sections: HmTCs embedded in the connective tissue, acquire the phenotype typical of migrating cells showing the cytoplasm filled with filament bundles perpendicularly oriented (**j**) (white arrowheads). Hemidesmosomes (**j**) (black arrowheads) are visible in connection with a thick basal membrane (BM). (**k**–**o**) HmTCs in angiogenic process: (**k**) semithin section of botryoidal (B) and endothelial cells shaping a new lumen (L) in which clusters of circulating precursor cells are visible (arrowhead). At ultrastructural level (**l**–**o**), HmTCs (some of them false-coloured in light blue) (**l**) are visible among circulating precursor cells (P) inside the neo-vessel (L: lumen). (**m**) Detail of relationship among branched HmTCs and circulating precursor cells (P). HmTC (**n**) with long telopode (arrowheads) in the capillary lumen (L). (**o**) HmTC “floating” (white arrowhead) in the lumen (L), is surrounded by numerous multivesicular bodies (black arrowheads). (**p**–**s**) Semithin and thin sections of leech wound healing. The regenerating tissue is characterised by a large amount of migrated cells. Among them, HmTCs (arrowheads) (**p**,**q**,**r**) engage numerous direct interaction with adjacent cells (**q**) and/or with collagen (**s**) via proteoglycans (white arrowheads). Close to HmTCs multivesicular bodies are visible (**q**,**r**) (black arrowheads).

Ultrastructural studies are considered the “gold standard” to identify the morphology of TCs (Fig. 1 panels j,k,n). TEM analysis are performed to identify easily and undoubtedly the characteristic features described in vertebrates. It can visualize the ovoidal cell body, where the scarce cytoplasm is occupied by a large nucleus and elements of cytoskeleton, and the thin telopodes (Fig. 1 panels j,k). These thread-shaped cytoplasmic processes showed the alternated tubular podomers and the enlarged podoms (Fig. 1 panel n) containing microsomes and lysosomes, attested by the intense non specific esterase (NSE) (Fig. 1 panel l) and mitochondria detectable according to the intense succinic dehydrogenase (SDH) reaction (Fig. 1 panel m). SEM, TEM and AFM images, demonstrated the three-dimensional network formed by interconnected HmTCs (Fig. 2 panels a–e) showing that HmTCs were mainly oriented to surround muscle fibers (Fig. 2 panels a–c) and to envelop the large collagenous bunches perpendicularly (Fig. 2 panels d,e).

Stimulated/activated HmTCs changed their behavior, moving towards the site of LPS or surgical lesion (Fig. 1 panel i). The HmTC conformational changes are functionally characteristic of progressive amoeboid feature (Fig. 2 panels f–j) and hemidesmosomes and bundles of cytoskeletal filaments are visible (Fig. 2 panel j).

In adult *H. medicinalis*, after injury, numerous vessels are newly formed, spanning throughout the formerly avascular muscular body wall, through two main steps. The first, evoking vertebrate vasculogenesis, is due to the remodeling of the cordonal botryoidal tissue that starting from a solid cord of cells through a dehiscence process, forms a tubular pre-vascular structure. During the subsequent angiogenic step, new capillaries outgrow and split from pre-existing vessels. Parallel to the development of neo-capillaries, clusters of leukocytes-like cells and HmTCs develop inside the growing vessel lumen^37,42,48^ (Fig. 2 panels k–o).

During the wound closure and restoration, HmTCs participate, together with various cell types, in regeneration of the structures disrupted by surgical or traumatic actions, increasing in number, and arranging themselves in prevalently parallel rows (Fig. 2 panels p–r). The complex granulation stage is also characterized by fibroplasia^40,41^ with massive transient synthesis of ECM components and deposition of packed collagen fibrils on which HmTCs were laid perpendicularly (Fig. 2 panels d,e). Microscopic analysis revealed that HmTCs were also connected with collagenic matrix via proteoglicans, detected by specific Cupromeronic Blue staining (Fig. 2 panel s). The HmTCs which are organized in a 3D network due to the interstitial position connect and influence numerous different cell types via direct contacts and by shedding vesicles and exosomes (Fig. 2 panels o,q,r).

#### Immunocytochemistry

Immunophenotype of HmTCs is quite complex because this type of cell can express a large array of receptors engaged in immune surveillance that can be constitutively expressed or induced or altered.

**Figure 3:**
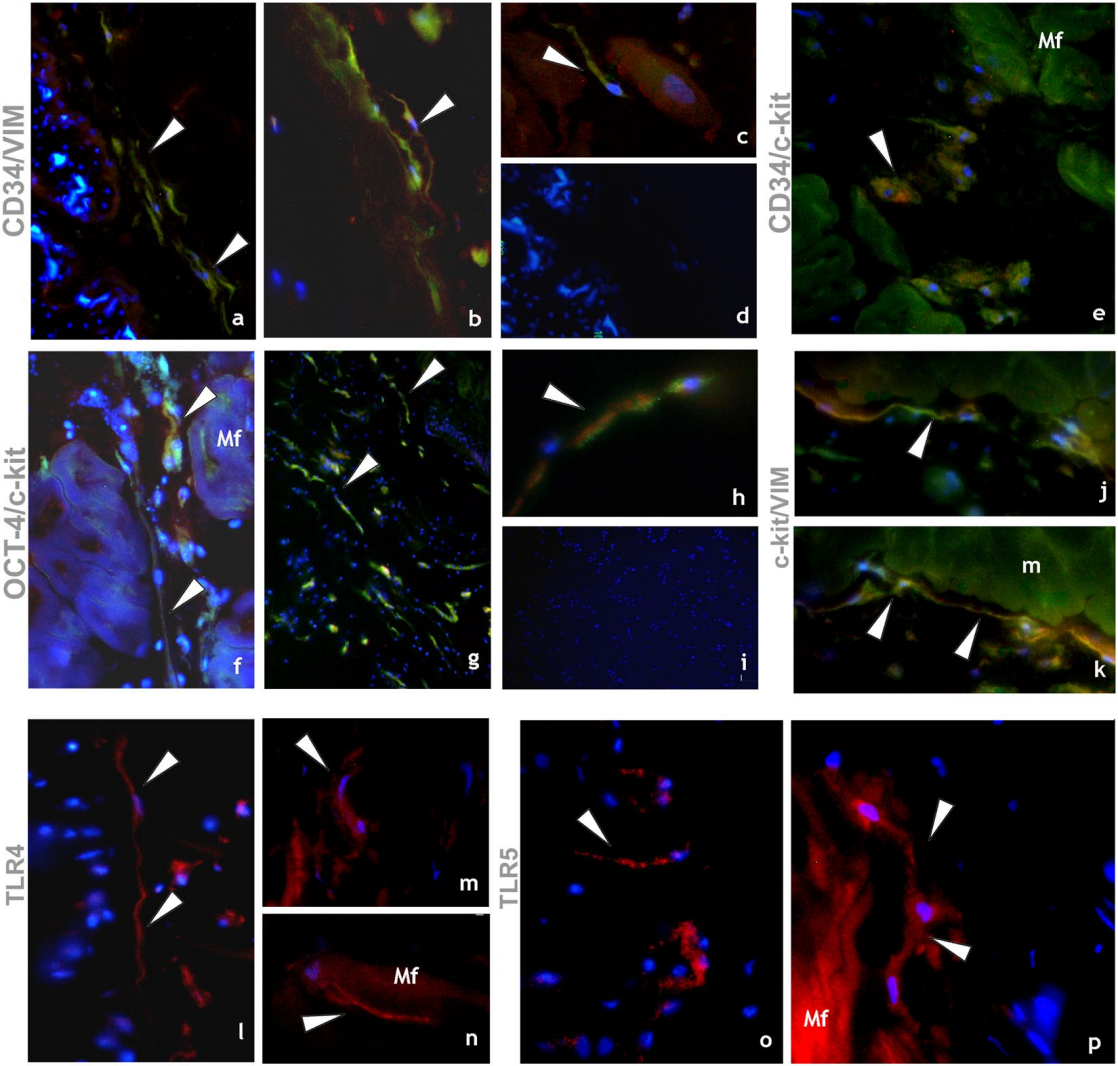
Immunocytochemical characterization of HmTCs. (**a**–**p**) Cryosections of leech activated body wall: (**a**–**k**) Double-immunostainings proposed as appropriate to identify telocytes. Samples stained by using antibodies against CD34 (green)/vimentin (red) (**a**,**d**); CD34 (green)/c-kit (red) (**e**); Oct-4 (green)/c-kit (red) (**f**–**h**); c-kit (green)/vimentin (red) (**j**,**k**) reveal cells with typical phenotype of telocytes (arrowheads). The two signals co-localize (merge in yellow) (arrowheads). (**d**,**i**) Negative controls. Nuclei are counterstained with DAPI and marked in brilliant blue. (**l**–**p**) HmTCs, wide spread in the body muscular sac (Mf), precisely identified for their morphology, are characterized by expression of TLRs (arrowheads). TLR4 (**l**–**n**) and TLR5 (**o**,**p**) that are evolutionarily conserved receptors playing a critical role in the early innate immune responses.

*Expression of markers typical of TCs*: HmTCs expressed constitutively a double-positive immunostaining with CD34/vimentin (Fig. 3 panels a-d), defined as appropriate for this type of cell in vertebrates as well as for CD34/c-kit, Oct-4/c-kit, c-kit/vimentin (stem cell markers expressed synchronously with the TC hallmarks) (Fig. 3 panels a–k).

*Markers of immuno-surveillance cellular system*: An antigenic profile was performed in order to identify the molecules that recognize the non-self and that consequently mediate the activation of innate immunity resulting in inflammation processes. HmTCs expressed Toll-like receptors (TLR) 4 and 5 (Fig. 3 panels l–p). Importantly, TLRs are present not only in immune cells such as circulating cells, monocytes, leukocytes and macrophages, but also in non-immune cells (fibroblasts, endothelial cells, adipocytes, epithelial cells and glial cells) and play a pivotal role in detecting invading pathogens and initiating a rapid immune response to them^50^. Specifically TLR4 recognizes bacterial LPS and endogenous molecules produced during tissue damage, while TLR5 recognizes the presence of Gram-positive and Gram-negative bacterial flagellin. The activation of TLRs, mobilizing the nuclear factor NF-kB, leads to the synthesis of a plethora of molecules such as pro-inflammatory cytokines (IL-18)^51^.

**Figure 4:**
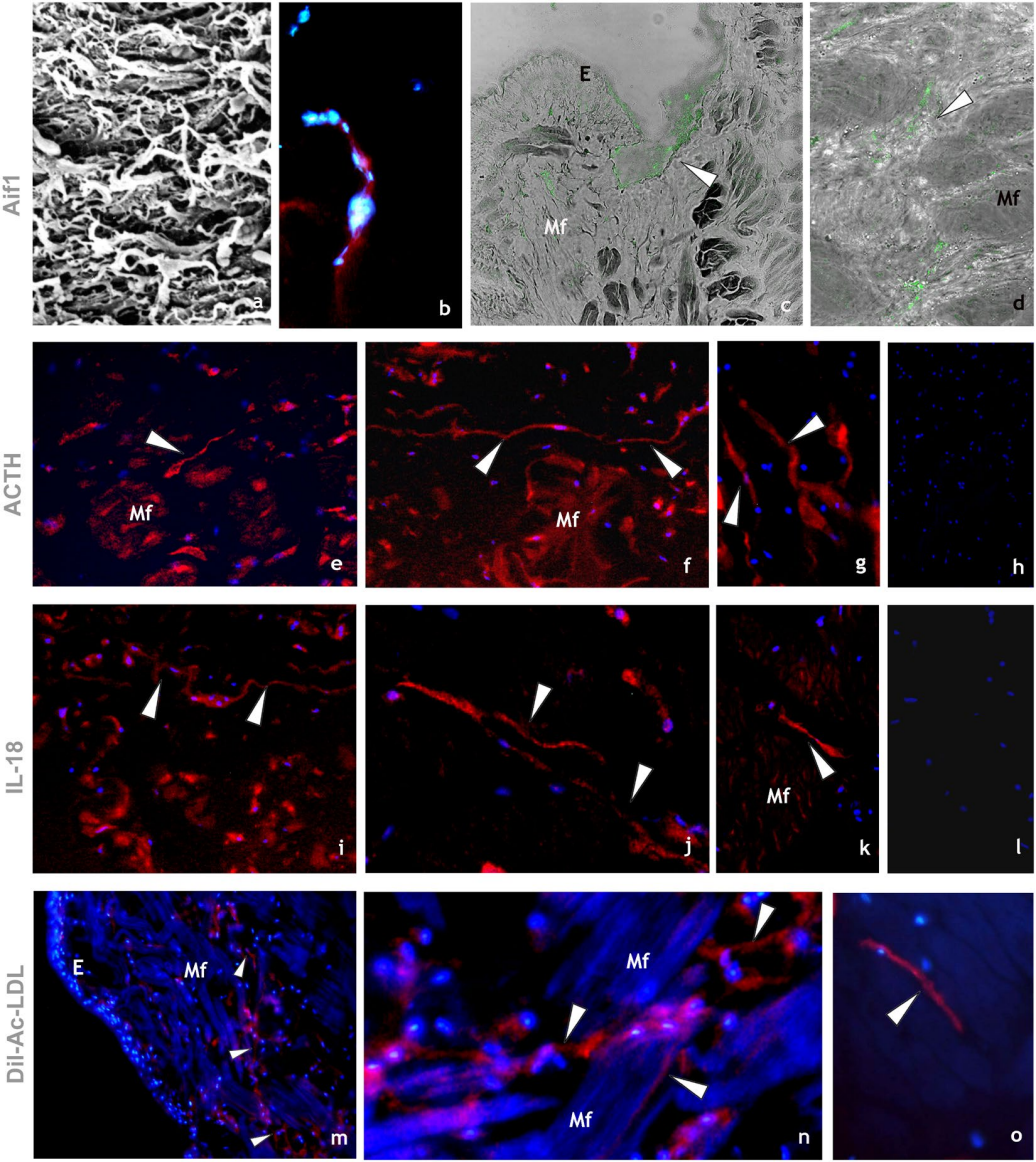
Immunocytochemical characterization of HmTCs. (**a**–**d**) HmTCs, recruited into lesioned area together with various cell types involved in migration and wound restoring, show own ultrastructural (SEM) profile (**a**). Cryosections of leech activated body wall: HmTCs are Aif-1 positive (red) (**b**). Nuclei are counterstained with DAPI (blue). (**c**,**d**) Immune-responsive cells (not exclusively belonging to telocytes) located under the epithelium (E) and among muscle fibres (Mf) are Aif-1 positive (green). Aif-1, also known as IBA1, is identified as a modulator in immune defence reaction and inflammatory response. (**e**–**g**) HmTCs (arrowheads) localized among muscle fibres (Mf), recognizable for their spindle-shaped body and long cellular prolongations, are ACTH positive. ACTH expression is signal of activation of stress-sensoring circuits. Not only telocytes positively bound antibody anti ACTH but these stromal cells are always identifiable for their morphology. (**h**) Negative control. Nuclei are stained with DAPI and marked in brilliant blue. (**i**–**k**) Cryosections of leech activated. HmTCs recognizable among various cell types (arrowheads) are IL-18 positive. The production of this representative pro-inflammatory cytokine is generally linked with activating innate immunity. (**l**) Negative control. Nuclei are stained with DAPI. (**m**–**o**) Cryosections of leech activated. DIL-ac-LDL uptake is considered a good tool to define the origin and the fate of hematopoietic progenitor cells. HmTCs, DIL-ac-LDL positive (arrowheads), can migrate from the lumen of neovessels (see Fig. 3 panels **k**–**o**), travelling in the connective tissue, among muscle fibers (Mf).

These multifunctional cells, wide-spread in the leech body, take part in regeneration of the structures disrupted by surgical lesion (Fig. 1 panel i; Fig. 4 panel a) also expressing HmAif-1, which plays a key role during inflammatory responses^52,53^ (Fig. 4 panels b–d).

*Immune and neuroendocrine system connections*: Gathering the evidence reported previously, we surmise that HmTCs, like many other invertebrate immunocytes, also play a role in the interconnection between the immune and neuroendocrine systems. In this context we demonstrated in HmTCs the expression of adrenocorticotropic hormone (ACTH) (Fig. 4 panels e–h) which is to be both an immune and neuroimmune molecule typical of vertebrates and invertebrates that performs specific responses strictly linked to inflammation and involved in hormone production.

It is well known that the cross-talk between the immune and neuroendocrine systems is also regulated by secretion of common chemical mediators such as cytokines^44,45,54,55^. For this reason, the expression of endogenous IL-18 was explored by an immunocytochemical analysis. HmTCs expressed this representative pro-inflammatory cytokine that is well known to activate innate immune responses in invertebrates and vertebrates^42,49,54,55^ (Fig. 4 panels i–l). IL-18 is originally identified as a leucocytic chemo-attractant factor also produced by numerous types of cells including immunocytes, lymphocytes and microglia^44,45,48^.

*Vital staining and cell identification*: To asses a relationship between vessel-associated circulating precursors and HmTCs and the fate of these cells during the regenerating process, vital Ac-LDL marker was utilized.

In vertebrates, Dil-Ac-LDL marker has been used to identify and characterize precursor cells and their destiny when they arrive in connective tissue. After injecting Dil-Ac-LDL in the leech body wall, HmTCs-DIL^+^ were found dispersed within the muscle interstitial space of body wall (Fig. 4 panels m–o).

## Discussion

The present paper provides evidence for the presence of TCs in the leech *H. medicinalis* which in this context is considered a good invertebrate animal model. This organism shows a “parenchimatous” body in which the loose connective tissue is colonized by several and different types of stromal cells such as macrophages, fibroblasts, myo-fibroblasts, NK-like cells and granulocytes, that share the same phenotype and function closely to the vertebrate counterpart. In addition, the basic steps of cellular responses, evoked by different stimuli, are strikingly similar to those observed in higher forms of life.

**Figure 5:**
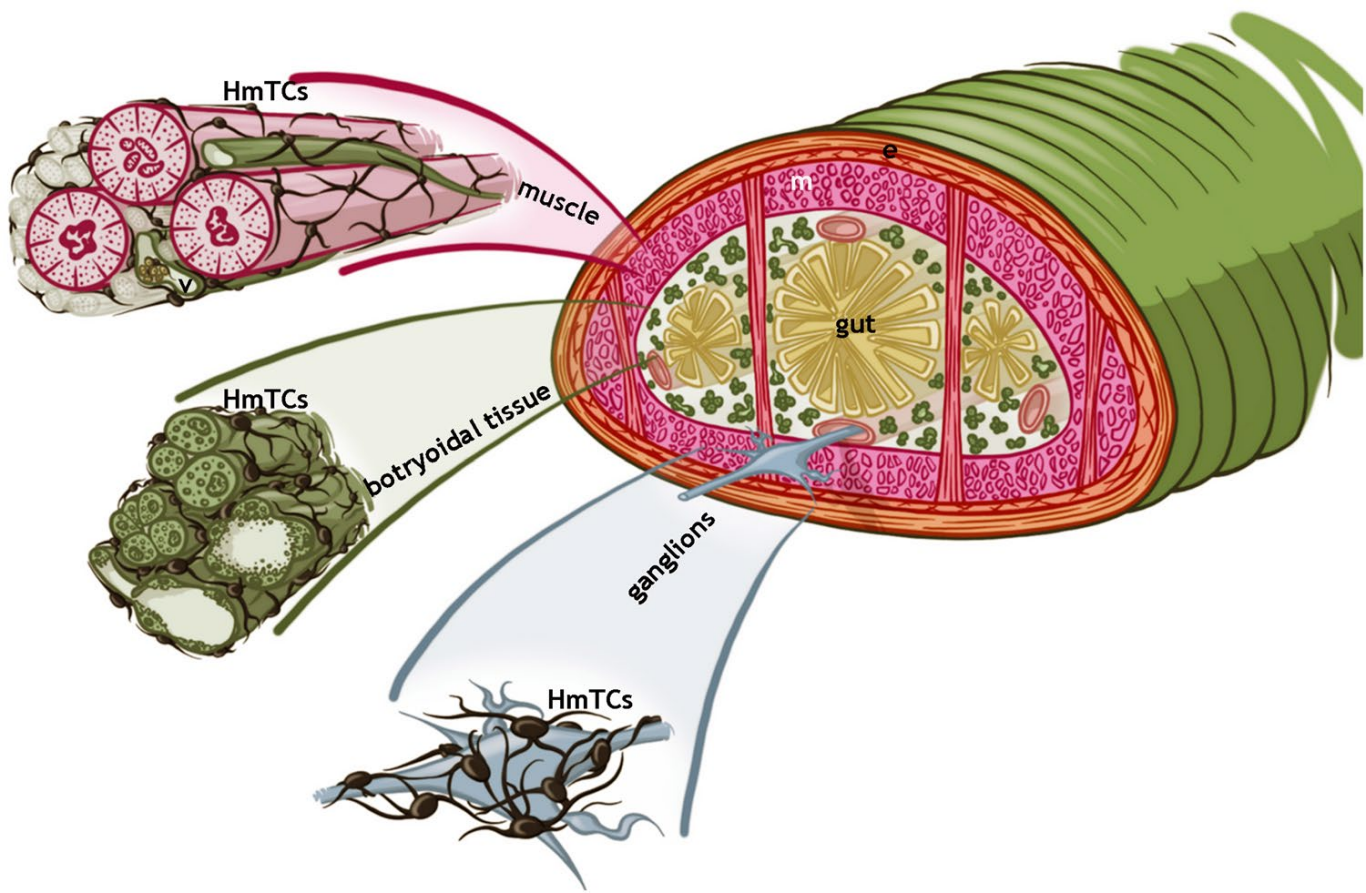
Schematic overview explaining the spatial organization of HmTCs in Hirudo medicinalis. Telocytes (HmTCs) forming a 3D network connect cells from muscular, botryoidal, nervous and connective tissues.

Among the leech stromal cells, the HmTCs can be detected due to their affinity for specific vital dyes. In non-stimulated animals, HmTCs in resting condition show the typical morphology described for vertebrate TCs: a pyriform or small ovoidal cell body with very long and thin cytoplasmic processes elongated up to 50 μm that are occupied by mitochondria, bunches of filaments perpendicularly oriented and few granules. The HmTC branches extend them in different directions and are engaged in cell-cell and cell-matrix contacts. The HmTCs form a network interconnecting cells from muscle layers, vessels, nervous system as well as collagenous bundles of connective tissue filling the leech body (Fig. 5, explanatory drawing). Once activated (after injection of LPS or following surgical lesion), these cells, organized in a plastic and dynamic system, can change their phenotype, acquiring an elongated shape suitable for migration with shorter telopods characterized by the involvement of the cytoskeletal elements.

HmTCs in healthy and in activated leeches are identified, as in vertebrate (i.e. in human) by double-positive immunostaining with CD34/vimentin, c-kit/vimentin, c-kit/CD34 and c-kit/Oct-4 which are stem cell markers expressed simultaneously with the hallmarks of TCs.

It is important to underline that another important characteristic of HmTCs is that they can recognize microbial components through TLRs that mediate activation of innate immunity. The detection of the non-self results in the inflammation phase with cell surface activation molecules followed by secretion of pro-inflammatory cytokines such as IL-18. These multifunctional cells, as several innate immune cells, express also HmAif-1, other factor typically involved in inflammatory responses^52,53^.

HmTCs, similarly to the major inflammatory cell types (i.e. immunocytes), are equipped to produce hormones such as ACTH and are able to respond to them. In our previous studies we have demonstrated that ACTH loop (controlling locally immune reactivity and regulating the cellular functions of the immune system in an autocrine and paracrine manner) has a key role in mutually influencing immune and neuroendocrine functions^44,45,56–60^.

The HmTC complex, that we defined as a “system in the system”, represents the most primitive immune organization, participating and conducting the first line of tissue/organism defences. The powerful ability of HmTC stromal network is due to the 3D organization that guarantees cross-talk among various cell types via intercellular contacts, secretion of cytokines and release of extracellular vesicles and exosomes.

These cells share with the various types of immunocytes the same mesodermal lineage as evidenced by their presence, after stimulation, inside the lumen of neo-vessels where are strictly connected with leukocyte-like cells. Experiments with the vital marker Dil-Ac-LDL, which is taken up preferentially by endothelial cells and precursor cells, are useful for staining and following the fate of these cellular types and to confirm the presence of HmTCs localized among precursors of circulating cells, within the lumen of newly formed vessels and dispersed among the groups of muscle cells or localized close to the injured region in wounded leeches. This finding reinforced the concept that hematopoietic precursor cells and telocytes, both of which develop in close association, have a mesenchymal common origin as in vertebrates.

Since HmTCs configure a 3D network of resident sentinels embedded in the connective tissue matrix, they can detect the first sign of non-self presence, and are required for proper communication among different cell types in all regions of the body. The HmTC immuno-surveillance system, obtained by direct cell-cell contacts and by soluble mediators released *in loco*, controls and directs the activity of neighbouring body cells. Once activated, the first response of HmTCs results in an alert centripetally and centrifugally directed. These communication precede the responses of integrated systems (i.e. cellular and soluble molecules) that the organism utilizes against any type of non-self. Moreover HmTC cross-talk by-passes both the slowness of the other types of innate immune cells that have to migrate and increase numerically before becoming operative and the slower spread, via matrix, of soluble molecules (growth factors and cytokines) that are produced by those cells directly involved in lesion or injected sites.

Thanks to the characteristics of stem cell, HmTCs are also engaged in regenerative processes at the lesion area.

Overall detailed analysis demonstrates that HmTCs are organized in a primitive ancient 3D system, part of innate immunity both in invertebrates and vertebrates. The fact that invertebrates and vertebrates could share cells, molecules and related functions inserted on a basic frame is not surprising according to an economic, conservative strategy in which a basic cellular network can provide the first line of body/tissue defence followed by more specific responses.

Moreover, due to the features of HmTC network we extend the list of leech defense mechanisms by addressing the problem of immunological memory in invertebrates. We have already suggested the existence of a kind of positive immune memory in allo- and xeno-transplantation in leeches^40^ and demonstrated that in second-set grafting experiments, responses to the second transplant were always faster and stronger than those occurring in first-set grafting experiments. Now we hypothesize that this form of innate immune memory (trained immunity) in invertebrate, unrelated to antibodies that mediate adaptive immunity, could be explained by how with the HmTCs’ function.

Indeed, a provocative thought arises from the intriguing comparison of HmTC network with microglia cells, not only in leeches but also in vertebrates. Interestingly microglia and TCs can be considered as the ancient conserved interface-system between immune and neuroendocrine function because share similar mediators and receptors from lower invertebrate up to human^61–67^. Support for this idea comes from comparative studies showing that TCs and microglia from *H. medicinalis* and vertebrates present a number of similarities in morphology, cellular functions, cell surface antigens, histochemical properties, mesodermal origin, capability to migrate, proliferate and differentiate in response to injury. It could be of interest to evaluate the possibility that TCs and microglial cells could represent the same type of cell resident in different districts able to detect the first signs of non-self invasion or tissue damage.

## Methods

### Animals and Treatments

Medicinal leeches [*Hirudo medicinalis* (Annelida, Hirudinea) from Ricarimpex, Eysines, France] were kept in tap water at 19–20 °C in aerated tanks. Animals were fed weekly with calf blood. Before each experiment, leeches were anesthetized with a 10% ethanol solution. Animals were subdivided into separate experimental groups (twenty animals/group) according to the treatments used. 1) uninjured leeches as control. 2) leeches subjected to surgical lesion affecting the entire thickness of the body wall at the hind part of the animal (80^th^ dorsal, superficial metamere). 3) leeches injected (at 80^th^ dorsal, superficial metamere) with lipopolysaccharide (LPS) (100ng/ml from *Escherichia coli*, serotype O55:B5, Sigma-Aldrich) used for immune stimulation.

Anesthetized leeches were killed, dissected and fixed 1 h after LPS stimulation and 1 week after surgical lesion.

All chemicals were purchased from Sigma (Italy) unless otherwise indicated.

### Light microscopy, transmission (TEM), and scanning (SEM) electron microscopy (Standard procedure)

Samples were collected and were fixed at 4 °C for 2 h in 2% glutaraldehyde in 0.1 M Na-cacodylate buffer (pH 7.2). Specimens washed in 0.1 M Na-cacodylate buffer (pH 7.2), were post-fixed at 4 °C with 1% osmic acid in cacodylate buffer (pH 7.2) for 2 h. After standard dehydration in ethanol series, samples were embedded in an Epon-Araldite 812 mixture and sectioned with a Reichert Ultracut S ultratome (Leica, Nussloch, Germany). Semithin sections were stained by conventional methods (crystal violet, methylene blue, May-Grünwald Giemsa and Toluidine blue stainings) and were observed with a light microscope (Olympus, Tokyo, Japan). Images were acquired with a Nikon DS-SM camera. For TEM, thin sections were stained by uranyl acetate and lead citrate and observed with a Jeol 1010 electron microscope (Jeol, Tokyo, Japan). Data were recorded with a MORADA digital camera system (Olympus).

For SEM, the dehydrated samples were treated with hexamethyldisilazane and mounted on polylysinated slides. The samples were air-dried and covered with a 9 nm gold film by flash evaporation of carbon and gold in an Emitech K250 sputter coater (Emitech, Baltimore, Md, USA). The specimens were examined with a SEM-FEG Philips XL-30 microscope (Philips, Eindhven, Netherlands).

### Atomic Force Microscope (AFM) procedure

Dehydrated specimens in hexamethyldisilazane, as previously described for SEM preparation, were observed in tapping-mode AFM (TMAFM) on a Digital Instruments Nano-scope III multimode microscope, fitted with Nanosensors SSS-NCH silicon probes (force constant, 30–60 N/m and resonance frequency, 300–350 kHz). All measurements were performed in air at a scan rate of approx. 2 Hz. To enhance the resolution of TMAFM images without compromising their accuracy and linearity, TMAFM files were rendered in three-dimensions by photorealistic ray-tracing rendering software (POV-RAY, version 3.0).

### Electron histochemistry

#### TEM Cupromeronic Blue procedure

Cupromeronic Blue is an intense blue cationic dye for ultrastructural localization and characterization of proteoglycans. Specimens were incubated overnight at 4 °C in 25 mM sodium acetate buffer, pH 5.8, containing Cupromeronic Blue (USBiological, Swampscott, Massachusetts, USA), 2.5% glutaraldehyde, and 0.1 M MgCl_2_.

Samples were dehydrated, embedded in an Epon-Araldite 812 mixture and sectioned as previously described in the paragraph concerning TEM standard procedures.

### SEM and TEM Osmium maceration procedure

This technique is utilized to highlight the three-dimensional architecture of extracellular and intracellular areas.

SEM analysis: samples of leech body cross-sections, were embedded in Polyfreeze tissue freezing medium (OCT, Polyscience, Eppelheim, Germany) and immediately frozen in liquid nitrogen. Cryosections (8–10 μm) were obtained with a Leica CM1850 cryotome and collected on gelatine-coated slides.

After washing in phosphate-buffered saline (PBS) (pH 7.2) for 5 min at room temperature, were postfixed in a solution of 1% osmium tetroxide and 1.25% potassium ferrocyanide for 30 min at room temperature. After washing in PBS (pH 7.2) for 5 min at room temperature, slides were immersed in 0.1% osmium tetroxide in PBS for 48 h at room temperature then processed for SEM observation.

TEM analysis: samples from cross-sections of leech body were fixed in a solution of 1% osmium tetroxide and 1.25% potassium ferrocyanide for 30 min at room temperature. After washing in PBS (pH 7.2) for 5 min at room temperature, specimens were immersed overnight in 0.1% osmium tetroxide in PBS at room temperature then processed for TEM observation.

### Immunohistochemistry

Ten untreated leeches that served as controls and ten injured leeches (subjected to deep lesions affecting the entire body wall) were utilized.

Samples from leech body cross-sections were embedded in Polyfreeze tissue freezing medium (Polysciences, Eppelheim, Germany) and immediately frozen in liquid nitrogen. Cryosections (7 μm) obtained with a Leica CM 1850 were utilized.

c-kit (CD117), CD34 (surface marker) and Oct-4 (transcription factor) as stem cell marker panel and the presence of vimentin (mesenchymal cell marker) have been identified to verify the status of HmTC stemness.

These markers were assessed using the following primary antibodies: polyclonal anti-c-kit (CD117) (1:50, Abcam, USA); monoclonal anti-CD34 (1:40, SantaCruz, USA); polyclonal anti-Oct-4 (1:100, Abcam, USA) and monoclonal anti-vimentin (1:50, Abcam, USA).

The presence of adrenocorticotropic hormone (ACTH) and of the pro-inflammatory interleukin 18 (IL-18) was assessed by using the following primary antibodies: polyclonal anti-ACTH (1:100 dilution, Abcam, Cambridge, UK); polyclonal anti-IL-18 (1:100 dilution, Abnova, Taipei City, Taiwan).

Toll-like receptors (TLRs) (to sense the presence of Danger Associated Molecular Pattern) were localized by using polyclonal anti-TLR4 and TLR5 (1:100, Abcam, USA).

HmAif-1, [homolog to human allograft inflammatory factor-1 (Aif-1) also known as IBA-1 protein that, in vertebrates, plays an important role in the allograft immune response to inflammation] was assessed using a polyclonal anti-HmAif-1 (1:100, New Zealand White, SPF) (Proteogenix, France)^67^.

Incubations with suitable secondary antibodies conjugated with Cyanine5 (Cy5; 1:50 dilution, Abcam) and with Fluorescein isothiocyanate (FITC; 1:200 dilution, Abcam) were performed for 1 h in a dark humid chamber at room temperature. Nuclei were stained with 4′,6′-diamino-2-phenylindole (DAPI). The PBS buffer used for antibody dilutions contained 2% bovine serum albumin (BSA) and 0,1% Tween 20. In co-localization experiments: -the sections were first stained with CD34 and then incubated with vimentin antibody or with c-kit; -the sections were first stained with c-kit and then incubated with vimentin or with Oct-4 antibodies (as described above).

In control samples, primary antibodies were omitted, and samples were treated with BSA/Tween20-containing PBS. Coverslips were mounted in citifluor (Citifluor Ltd, London, UK). Slides were observed either under an Eclipse Nikon microscope or Leica laser confocal microscope (Leica Microsystems, Germany).

### *In vivo* tracking experiments with Acetylated Low Density Lipoprotein, labelled with 1,1\‘-dioctadecyl – 3,3,3\‘,3\‘-tetramethyl-indocarbocyanine perchlorate (DiI-Ac-LDL)

Five untreated leeches, five leeches injected with 100 μl of PBS and five injured leeches (subjected to deep lesions affecting the entire body wall) were injected with 10 μl of a Dil-Ac-LDL (Biomedical Technologies Inc., MA, USA) solution [(10 mg/ml in phosphate buffer (PBS)], the vital marker specific for circulating precursor cells and endothelial cells. After 4 h (corresponding to a suitable time necessary for the dye to diffuse in the body wall of the animal) the leeches were sacrificed and dissected according to the manufacturer’s guideline. Samples were embedded in Polyfreeze tissue freezing medium (Polysciences, Eppelheim, Germany) and immediately frozen in liquid nitrogen. Cryosections (7 µm) obtained with a Leica CM 1850 were utilized to identify cells Dil+. Dil-Ac-LDL uptake was visualized on a fluorescence microscope Olympus BH2 through a rhodamine filter set (excitation/emission filters 550/580 nm). Images were acquired with a DS-5M-L1 Nikon digital camera system.

When endothelial and precursor cells are labelled with DiI-Ac-LDL, the lipoprotein is degraded by lysosomal enzymes and the DiI (fluorescent probe) accumulates in the intracellular membranes. By employing Dil intense fluorescence, fine structures such as telopods of the TCs can also be visualized in leech tissues. Labeling with DiI-Ac-LDL offers several advantages; first because once the cells are labeled, the fluorescent probe (DiI) is not removed and also because no other cell type such as fibroblasts, smooth muscle, pericytes, epithelial cells are labelled as vascular endothelial and precursor cells.

### Histochemistry

Frozen cross-sections (10 μm) of five surgical stimulated leeches were stained, according to manufacturer’s instructions, using a histoenzimatic kit, (Bio-optica, Milan, Italy) to test for Succinic dehydrogenase (SDH), attributable to mitochondrial activity and Non specific Esterase (NSE) to localize microsomal and lysosomal activity.
